# RNA∶DNA Hybrids Initiate Quasi-Palindrome-Associated Mutations in Highly Transcribed Yeast DNA

**DOI:** 10.1371/journal.pgen.1003924

**Published:** 2013-11-07

**Authors:** Nayun Kim, Jang-Eun Cho, Yue C. Li, Sue Jinks-Robertson

**Affiliations:** Department of Molecular Genetics and Microbiology, Duke University Medical Center, Durham, North Carolina, United States of America; CABIMER, Universidad de Sevilla, Spain

## Abstract

RNase H enzymes promote genetic stability by degrading aberrant RNA∶DNA hybrids and by removing ribonucleotide monophosphates (rNMPs) that are present in duplex DNA. Here, we report that loss of RNase H2 in yeast is associated with mutations that extend identity between the arms of imperfect inverted repeats (quasi-palindromes or QPs), a mutation type generally attributed to a template switch during DNA synthesis. QP events were detected using frameshift-reversion assays and were only observed under conditions of high transcription. In striking contrast to transcription-associated short deletions that also are detected by these assays, QP events do not require Top1 activity. QP mutation rates are strongly affected by the direction of DNA replication and, in contrast to their elevation in the absence of RNase H2, are reduced when RNase H1 is additionally eliminated. Finally, transcription-associated QP events are limited by components of the nucleotide excision repair pathway and are promoted by translesion synthesis DNA polymerases. We suggest that QP mutations reflect either a transcription-associated perturbation of Okazaki-fragment processing, or the use of a nascent transcript to resume replication following a transcription-replication conflict.

## Author Summary

Mutation rates are correlated with the level of gene expression in budding yeast, demonstrating a link between transcription and stability of the underlying DNA template. In the current work, we describe a novel type of transcription-associated mutation that converts imperfect inverted repeats (quasi-palindromes or QPs) to perfect inverted repeats. Using appropriate mutation reporters, we demonstrate that QP mutations are strongly affected by the direction of DNA replication and have distinctive genetic requirements. Most notably, rates of transcription-associated QP events are regulated by the RNase H class of enzymes, which are specialized to process the RNA component of RNA∶DNA hybrids. The source of the RNA∶DNA hybrids that initiate QP mutations is unclear, but could reflect transcripts that remain stably base-paired with the DNA template, or aberrant processing of the RNA primers normally used to initiate DNA synthesis. These studies further expand the diverse ways that transcription affects the mutation landscape, and establish a novel way that RNA∶DNA hybrids can contribute to genetic instability. The high conservation of basic DNA-related metabolic processes suggests that results in yeast will be broadly applicable in higher eukaryotes.

## Introduction

RNA∶DNA hybrids exist as normal intermediates during the cellular transactions of transcription and DNA replication. During transcription, a small segment of the nascent RNA is transiently base-paired with the template DNA strand as part of a transcription bubble. More extensive and stable hybrids between RNA transcripts and the DNA template (an R-loop), however, can form under certain conditions (reviewed in [1], [2], [3]). Impediments to the transcription process such as disruption of co-transcriptional mRNA packaging or a failure to remove negative superhelical stress in the transcribed region favor the formation of R-loops. RNA∶DNA hybrids also form during genome replication, especially during synthesis of the lagging strand, which occurs discontinuously as a series of ∼200 nt Okazaki fragments (reviewed in [4], [5]). Each Okazaki fragment is initiated by a complex of DNA polymerase α (Pol α) and primase, which together synthesize primers comprised of ∼10 ribonucleotide monophosphates followed by ∼20 deoxyribonucleotide monophosphates (rNMPs and dNMPs, respectively). Following a polymerase switch to Pol δ, the primary lagging-strand polymerase, primers are extended to complete Okazaki-fragment synthesis. Finally, RNA primers are removed by Pol δ-mediated strand displacement coupled with flap processing.

In addition to forming in association with transcription and replication, RNA∶DNA hybrids can also arise from the stochastic incorporation of rNMPs by DNA polymerases. Replicative DNA polymerases are highly selective in discriminating between rNTP and dNTP substrates, but nevertheless utilize rNTPs at a low level during DNA synthesis. In *Saccharomyces cerevisiae*, it has been estimated that Pol δ and Pol ε incorporate 1 rNMP for every 1,250 or 5,000 dNMPs, respectively [6]. In combination with Pol α, which has lower sugar discrimination than either Pol δ or Pol ε, there likely are >10,000 rNMPs incorporated during each round of haploid genome duplication. Though this generally is assumed to result in single rNMPs embedded in otherwise duplex DNA, tandem rNMPs could also be incorporated. In mammals, rNMPs are found at more than 1,000,000 sites per genome, suggesting that rNMPs are the most abundant type of endogenous DNA “lesion” [7].

RNA∶DNA hybrids are a serious threat to genome stability and are removed by the RNase H class of enzymes, which specifically degrade the RNA component (reviewed in [8]). RNase H1 is a single-subunit enzyme; it requires at least four contiguous rNMPs for cleavage of RNA∶DNA hybrids *in vitro* [9], [10] and is essential for the replication of mitochondrial DNA [11]. RNase H2 is a three-subunit enzyme that is highly expressed in proliferative mammalian tissues [7] and, in addition to its ability to process R-loops and Okazaki fragments, has the unique ability to incise at a single rNMP within otherwise duplex DNA [10], [12], [13]. Persistent R-loops interfere with replication during mitosis and meiosis, leading to accumulation of DNA breaks, especially at highly transcribed and/or repetitive sequences (reviewed in [1]). A failure to remove lagging-strand primers and complete Okazaki-fragment maturation likewise results in hyper-recombination, chromosomal instability, hyper-sensitivity to DNA damaging agents and reduced cell viability [14]. In the absence of RNase H2, accumulation of single (or a small number of) rNMPs in genomic DNA also leads to cell-cycle defects and chromosomal instability [7], [15]. Though neither RNase H1 nor H2 is essential in yeast, both are required for mammalian cell viability and embryonic development [7], [11]. Significantly, hypomorphic alleles of genes encoding RNase H2 subunits are associated with the hereditary, early-onset neuro-inflammatory disease Aicardi-Goutières syndrome [16].

In addition to gross chromosomal effects, persistent rNMPs in DNA elevate mutagenesis. There is a dramatic increase in deletions occurring within short tandem repeats in yeast strains deficient in RNase H2 [17], [18], [19], and a similar mutation signature is found in highly transcribed sequences [20]. The deletions in both cases require activity of the yeast Type 1B topoisomerase (Top1), an enzyme that relieves transcription-associated torsional stress in DNA (reviewed in [21]). The first step in a Top1-catalyzed reaction is generation of a transient single-strand break in the DNA backbone, with the enzyme linking itself to the 3′ end of the break via a covalent phosphotyrosyl bond. Following rotation of the broken strand around the intact strand, the Top1-generated nick is resealed and the enzyme is released in a self-catalytic reaction with the hydroxyl group on the 5′ side of the break. When the initial Top1 cleavage occurs at an rNMP, however, the 2′ hydroxyl group of the ribose can attack the phosphotyrosyl bond. This results in release of Top1 from DNA and leaves behind a nick flanked by a 2′,3′ cyclic phosphate and a 5′-OH [22]. We have proposed that when Top1 cleaves at an rNMP, either further processing of the dirty ends thus created or a subsequent cleavage-ligation cycle by Top1 results in short deletions [19]. The significance of the tandem repeat in the deletion process is that it provides an opportunity for strand misalignment, which converts a gapped intermediate into a more efficiently ligated nick. That rNMP-dependent deletions reflect Top1 incision at a single rNMP was recently confirmed using a mutant RNase H2 that retains only the ability to nick at tandem rNMPs [10].

In the current study, we describe a new type of transcription-associated mutation that is greatly elevated in RNase H2-deficient yeast strains. The relevant mutations occur at imperfect inverted repeats or quasi-palindromes and, in striking contrast to deletions in tandem repeats, do not require Top1 activity. Genetic analyses demonstrate a strong effect of the direction of replication fork movement as well as roles for translesion synthesis DNA polymerases and RNase H1 in generating these novel, Top1-independent events. We suggest that, in absence of functional RNase H2, RNase H1 generates potentially mutagenic roadblocks at sites of incompletely processed RNA∶DNA hybrids.

## Results

### Frameshift reversion assays

In order to obtain a variety of rNMP-dependent mutations, the reversion of three frameshift alleles that query an ∼150 bp sequence was monitored: *lys2ΔBgl, lys2ΔA746* and *lys2ΔA746,NR*. The *lys2ΔBgl* allele was constructed by filling in *Bgl*II-generated 5′ overhangs to generate a 4-bp duplication, the equivalent of a +1 frameshift mutation [23]. Reversion to lysine prototrophy requires acquisition of a compensatory net −1 frameshift mutation within a window defined by stop codons in the alternative reading frames. The *lys2ΔA746* allele contains an engineered 1-bp deletion in the same region of *LYS2* and requires a compensatory net +1 frameshift mutation for reversion [24]. Finally, the *lys2ΔA746,NR* allele was derived from the *lys2ΔA746* allele by disrupting mononucleotide runs in the reversion window that were longer than 3 bp [25]. For the *lys2ΔA746* and *lys2ΔA746,NR* alleles, transcription was driven by either the endogenous *LYS2* promoter or by a tetracycline/doxycycline-repressible promoter (*pTET*) to achieve low- or high-transcription conditions, respectively. For the *lys2ΔBgl* allele, the *pTET* promoter was fully active in the absence of doxycycline or was repressed to a low level by addition of doxycycline to the growth medium.

### Transcription-associated mutations accumulate at a quasi-palindrome in the absence of RNase H2 and are Top1 independent

**Figure 1:**
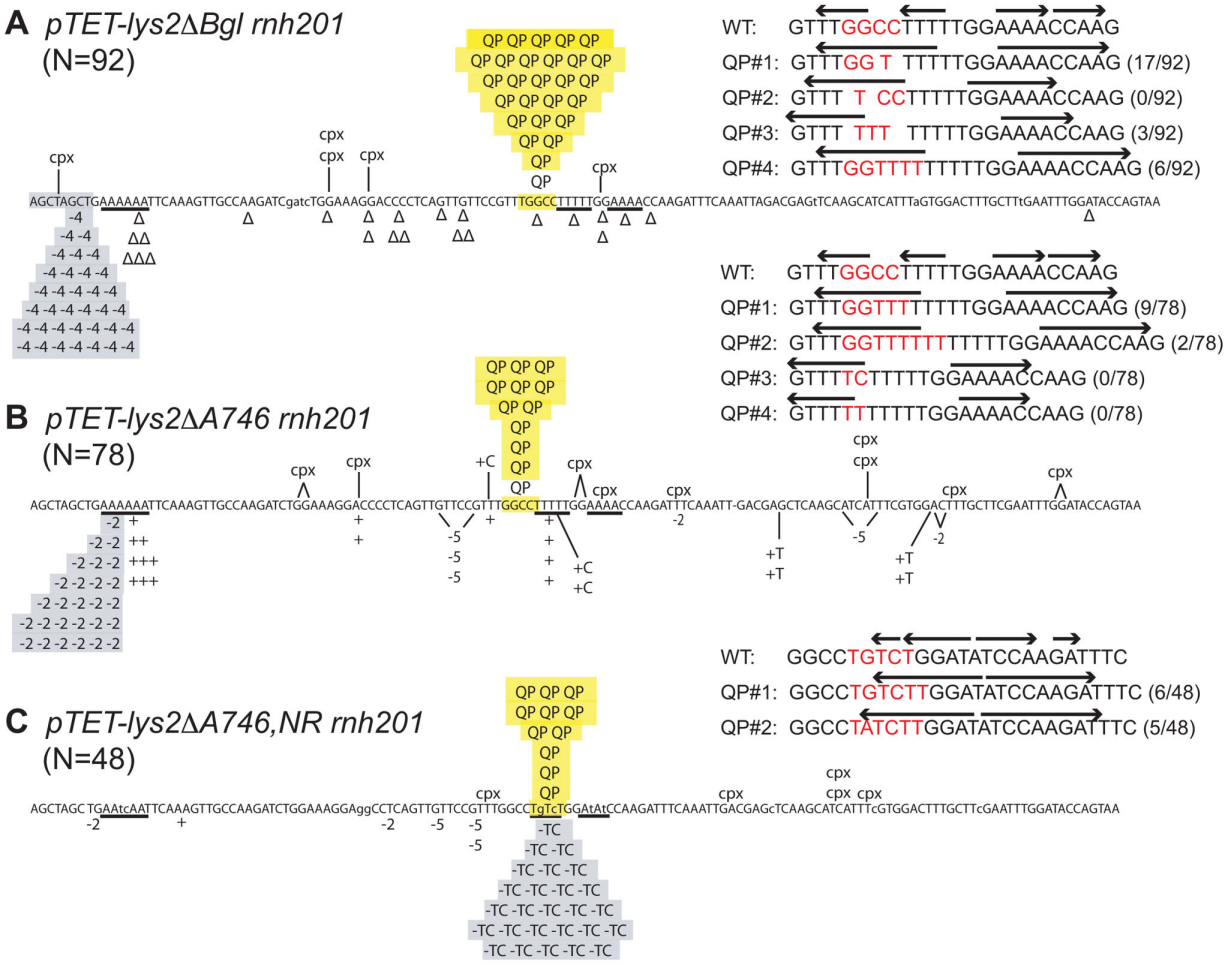
Reversion spectra in *rnh201Δ* backgrounds under high-transcription conditions. **A.** *pTET-lys2ΔBgl* revertants. **B.** *pTET-lys2ΔA746* revertants. **C.** *pTET-lys2ΔA746,NR* revertants. In each spectrum, 2- or 4-bp deletions at the Top1-dependent hotspot are highlighted gray; QP mutations at the Top1-independent hotspot are highlighted yellow. “N” refers to the number of Lys^+^ revertants sequenced for each spectrum; “Δ” and “+” signify a 1-bp deletion and insertion, respectively; cpx represents sporadic complex events that are not at the QP hotspot. The types and proportions of QP events at the hotspot in each spectrum are indicated. Arrows above the sequences correspond to inverted repeats; red bases indicate the positions and types of sequence changes in the QP events. It should be noted that some of the QP mutation types were not identified in the spectra shown and only became prominent when additional mutations were introduced. The proportions of QP mutation types identified in other genetic backgrounds are provided in Tables S1, S2, S3.

Loss of RNase H2 under low-transcription conditions is associated with only small changes in mutation rate, but can be accompanied by striking changes in mutation spectra [17], [26]. Mutation types that are specifically enriched in the absence of Rnh201, the catalytic subunit of RNase H2, will be designated here as rNMP-dependent mutations. In the *lys2ΔBgl* assay, RNase H2 loss is associated with reversion via deletion of one copy of an (AGCT)_2_ tandem repeat [26]. We previously demonstrated that the rate of this rNMP-dependent, 4-bp deletion (highlighted gray in Figure 1A) is greatly elevated under high-transcription conditions and that it requires the presence of Top1 [18], [19]. Specifically under high-transcription conditions, disruption of RNase H2 also resulted in a 100-fold increase in “complex” events, which are defined as mutations with multiple changes. These events occurred at a discrete hotspot (highlighted yellow in Figure 1A; see Table 1 for rates) and, in striking contrast to 4-bp deletions, their rates were not affected by Top1 loss (Table 1).

**Table 1:** Rates of rNMP-associated deletions and QP mutations.

*lys2* allele	Txn	Relevant	Mutation rate (×10^−10^)		
		genotype	Total Lys^+^ (95% CI)	2- or 4-bp deletion hotspot	QP hotspot
*lys2ΔBgl*	Low	WT	31.9 (26.3–47.3)	2.2	<0.44*
		*rnh201Δ*	68.8 (44.3–120)	56	<0.82*
	High	WT	784 (586–1310)	10	<10*
		*rnh201Δ*	3060 (2300–5790)	1160	865
		*rnh201Δ top1Δ*	2080 (1390–2990)	<23*	631
*lys2ΔA746*	Low	WT	18.4 (15.1–27.6)	<0.25*	<0.25*
		*rnh201Δ*	33.4 (21.2–59.3)	9.5	<0.37*
	High	WT	428 (335–674)	62	<3.7*
		*rnh201Δ*	3530 (2700–4090)	1220	499
		*rnh201Δ top1Δ*	1420 (1120–1670)	<16*	326
*lys2ΔA746,NR*	Low	WT	9.5 (8.6–10.4)	0.11	<0.11*
		*rnh201Δ*	15.3 (11.7–17.9)	6.9	0.34
	High	WT	161 (118–216)	1.9	<1.9*
		*rnh201Δ*	1470 (1210–1710)	796	337
		*rnh201Δ top1Δ*	812 (486–1370)	<17*	338

* No events observed; the rate was calculated assuming one event.

All *lys2* alleles are in the SAME orientation with respect to ARS306; CI, confidence interval.

Figure 1A shows sequence changes in four types of rNMP-dependent complex mutations at the hotspot detected in the *lys2ΔBgl* assay, each of which replaces all or part of a common GGCC sequence with one or more thymines. Significantly, this region contains two 4-bp inverted repeats (IRs) that together comprise a larger, imperfect IR or quasi-palindrome (QP). On the nontranscribed (coding) strand, the upstream segment of the QP is 5′ TTGGccTTTT (the lowercase letters are spacers between the smaller IRs), and the downstream segment is 5′-AAAACCAA; the two arms of the QP are separated by 3 bp. Each of the complex mutations generated a perfect IR that ranges in size from 5–8 bp, with the most frequent event generating an 8-bp palindrome. Hereafter, the transcription- and rNMP-dependent mutations originally designated as complex events will be referred to as QP mutations.

In the *lys2ΔA746* assay, *RNH201* deletion elevated the reversion rate 1.8-fold under low-transcription conditions (Table 1; [19]) and 2-bp deletions within a 6A run comprised ∼30% of reversion events (highlighted gray in Figure 1B). Under high-transcription conditions, 2-bp deletions at the 6A hotspot were prominent even in the presence of RNase H2, and were elevated an additional 20-fold in an *rnh201Δ* background. Like the 4-bp deletions detected in the *lys2ΔBgl* assay, 2-bp deletions in the 6A run were Top1 dependent. Also observed specifically under high-transcription conditions in the *rnh201Δ* background were QP mutations that localized to the same region as those in the *lys2ΔBgl* assay (highlighted yellow in Figure 1B). As in the *lys2ΔBgl* assay, QP mutation rates in the *lys2ΔA746* assay were not reduced when *TOP1* was deleted from the *rnh201Δ* background.

In the case of the *lys2ΔA746,NR* allele, deletion of *RNH201* under low-transcription conditions was accompanied by the appearance of a novel 2-bp deletion hotspot in which TC was deleted from the imperfect dinucleotide repeat, TGTCTG (events are highlighted gray in Figure 1C; see Table 1). The TGTCTG repeat is unique to the *lys2ΔA746,NR* allele and was generated during elimination of a 5T run (TTTTT changed to TgTcT) that overlaps the QP mutation hotspot in the *lys2ΔA746* and *lys2ΔBgl* assays. The TC deletions were elevated an additional 100-fold by high transcription, and like the short deletions in perfect tandem repeats, were dependent on Top1. In addition to TC deletions in the TGTCTG repeat, there were two types of rNMP-dependent +1 frameshift events at the same location, and these were observed only under high-transcription conditions (highlighted yellow in Figure 1C). One involved insertion of a T into the imperfect tandem repeat (TGTCTG changed to TGTCtTG), while the other was comprised of the same T insertion plus a nearby G to A transition (TGTCTG changed to TaTCtTG). Although the primary sequence of the *lys2ΔA746,NR* allele is slightly different from that of the *lys2ΔBgl* and *lys2ΔA746* alleles, a similar pattern of converting a QP to a perfect palindrome was evident. In this case, the arms of the QP are 5′-TCTGGAT and 5′-ATCCAaGA (the lowercase letter interrupts the palindrome); addition of a T (complementary to the disrupting “a” in the downstream arm) with or without an accompanying base change generates a perfect 8- or 9-bp palindrome, respectively (Figure 1C). As observed in the *lys2ΔBgl* and *lys2ΔA746* assays, QP mutations detected in the *lys2ΔA746,NR* assay were not affected by Top1 loss (Table 1). In summary, each frameshift reversion assay identified two distinct types of rNMP- and transcription-dependent mutations: Top1-dependent deletions in tandem repeats and Top1-independent events in quasi-palindromes.

### QP mutations are decreased when RNase H1 is eliminated

**Figure 2:**
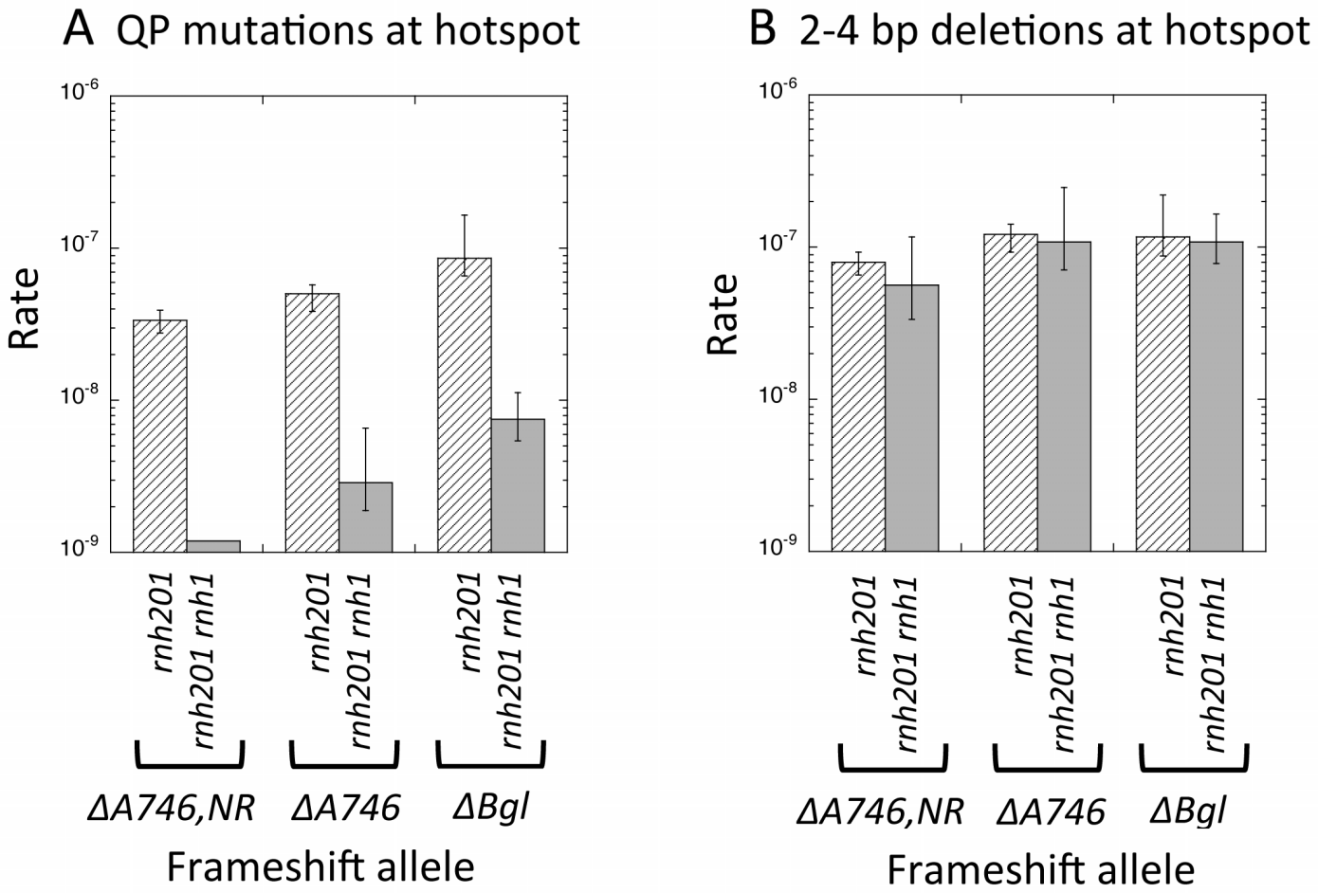
Rates of QP mutations and tandem-repeat deletions at hotspots in RNaseH1-defective strains. **A.** QP mutation rates at the common hotspot present in all three frameshift-reversion assays. **B.** Short deletion rates at the unique Top1-dependent hotspot identified in each frameshift-reversion assay.

The potential *in vivo* substrates of RNase H2 include rNMPs mis-incorporated into duplex DNA [17], the RNA primers at the 5′ ends of Okazaki fragments ([27], [28] and reviewed in [5]) and the RNA component of R-loops [8]. Though RNase H1 can neither incise at single rNMPs embedded in DNA [10] nor substitute for RNase H2 in a reconstituted ribonucleotide excision repair assay [13], it does share the other two types of RNA∶DNA substrates with RNase H2. To ascertain the type of RNA∶DNA hybrid that is relevant to QP mutations, we examined the effect of eliminating RNase H1 in addition to RNase H2. If only one or a few rNMPs is relevant to QP mutations, then disruption of RNase H1 in an *rnh201Δ* background would be expected to have no effect on these events. If it is multiple rNMPs that are relevant, however, then QP mutations should be further elevated upon additional loss of RNase H1. Unexpectedly, the rates of QP mutations in each frameshift-reversion assay were reduced at least 10-fold in the *rnh201Δ rnh1Δ* double- relative to the *rnh201Δ* single-mutant background (Figure 2A). As expected, the rates of Top1-dependent deletions at the tandem-repeat hotspots were not affected by additional deletion of *RNH1* in an *rnh201Δ* background (Figure 2B).

The opposing effects of RNase H1 and RNase H2 loss on QP mutations (reducing and enhancing these events, respectively) indicate that extensive RNA∶DNA hybrids, as well the few rNMPs that may remain after their processing, are relevant. Studies using mutant forms of Pol2 that have altered rNTP discrimination suggest that most rNMPs in yeast genomic DNA are incorporated by Pol ε, the leading-strand polymerase. To explore whether Pol ε might be the source of the rNMPs that initiate transcription-associated QP mutations, we introduced the rNTP-restrictive Pol2-M644L protein [6] into an *rnh201Δ top1Δ* background. Expression of the Pol2-M644L protein had no effect on the rates of QP mutations in either the *lys2ΔA746,NR* or *lys2ΔA746* assay (Tables S1, S2). These data suggest that the rNMPs that trigger QP mutations do not reflect aberrant incorporation by Pol ε, and likely are derived either from Okazaki fragment primers or from R-loops.

### QP mutations are affected by the direction of DNA replication

**Figure 3:**
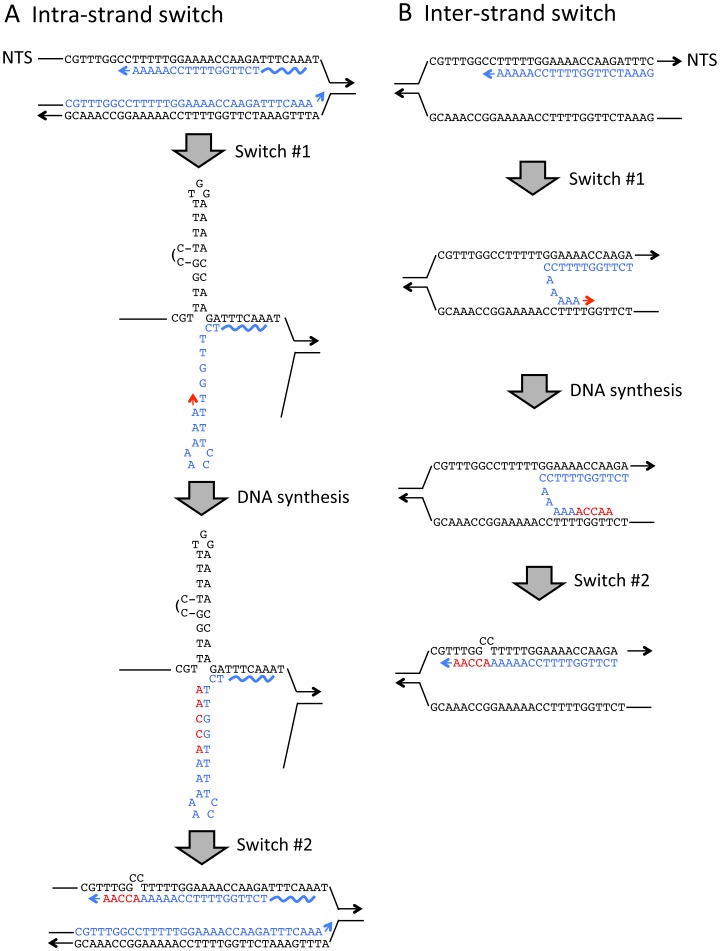
Strand switch models for generating QP mutations. The QP mutation illustrated is the most frequent event observed with the *lys2ΔBgl* allele (QP #1 in Figure 1A) and can be generated (**A**) via an intra-strand switch that occurs during lagging-strand synthesis or (**B**) when the nascent leading strand switches to the lagging-strand template. Arrowheads correspond to 3′ ends of DNA strands and the NTS strand of *LYS2* is indicated. Template bases are black; bases incorporated before the first template switch are in blue; and DNA synthesized after the template switch is red. The wavy line corresponds to an RNA primer on the lagging strand.

QP mutations have been proposed to occur via a template switch between the two arms of the QP during DNA synthesis (for reviews, see [29], [30]). In principle, this can involve either an intra- or an inter-molecular switch, each of which is illustrated in Figure 3 for the most common QP event observed in the *lys2ΔBgl* assay. Intra-molecular switches are thought to occur primarily during lagging-strand synthesis, where the single-strand nature of the template strand may contribute to hairpin formation and promote the initial template switch [31]. In the case of inter-molecular switches, data suggest that the nascent leading strand switches to the lagging-strand template, a switch that similarly may be facilitated by the extensive tracts of single-strand DNA formed between Okazaki fragments [32]. In either case, there are two switches: the first to an alternative template that is used to direct very limited DNA synthesis, and a second switch back to the original template.

Because the rate of a given QP mutation is often affected by the direction of DNA replication, it has been suggested that the corresponding intermediate is generated primarily during either leading-or lagging-strand synthesis [32], [33]. In all of the strains used here, the resident *LYS2* locus on Chromosome II was deleted and *lys2* frameshift alleles were positioned on Chromosome III near the early-firing replication origin *ARS306* [34]. When in the SAME orientation, which is the orientation used to generate the data in Table 1 and Figure 1, the *lys2* allele is oriented so that transcription from *pTET* is co-directional with the direction of replication fork movement from *ARS306*. In this orientation, the non-transcribed strand (NTS) of *lys2* is the lagging-strand template during DNA replication (Figure 3A). When in the OPPO orientation, the transcription and replication forks converge and the NTS strand is the leading-strand template (Figure 3B). To determine whether the direction of replication fork movement affects rNMP-dependent QP mutations, we compared their rates in SAME and OPPO strains. The direction of replication had a profound effect on QP mutation rates in each assay; when *lys2* alleles were in the SAME orientation, rates were at least 10-fold higher than when in the OPPO orientation (Table 2). As reported previously, the direction of DNA replication had no effect on overall levels of transcription-associated mutagenesis in a WT background, and only a subtle (at most 2-fold) effect on Top1-dependent deletions in tandem repeats (Table 2).

**Table 2:** Effect of replication direction relative to transcription on mutation rates.

		Mutation rate (×10^−10^)			
*lys2* allele	Relevant	2- or-4 bp deletions		QP mutations	
	genotype	SAME	OPPO	SAME	OPPO
*lys2ΔBgl*	*WT*	10.2	9.7	<10*	<9.7*
	*rnh201Δ*	1160	1500	865	<29*
	*rnh201Δ top1Δ*	<23*	<19*	631	<19**
*lys2ΔA746*	*WT*	62.2	54.1	<3.7*	<3.6*
	*rnh201Δ*	1220	689	499	48
	*rnh201Δ top1Δ*	<16.3*	7.8	326	31
*lys2ΔA746,NR*	*WT*	<1.9*	<1.6*	<1.9*	<1.6*
	*rnh201Δ*	796	394	337	<12*
	*rnh201Δ top1Δ*	<18*	<8.9*	338	36

All rates were measured under high-transcription conditions. Total Lys^+^ rates and the associated 95% confidence intervals are provided in Supplemental Tables.

* No events were observed; rate calculated assuming one event.

The sensitivity of QP mutations to the direction of DNA replication is consistent with strand switching that is triggered preferentially during either leading- or lagging-strand synthesis. Indeed, QP mutations in the *E. coli* genome are greatly elevated in the absence of mismatch repair (MMR), consistent with temporal connection of these events to replication fork movement [35]. To assess whether transcription-associated QP mutations in yeast are similarly limited by the MMR system, we examined reversion of the *lys2ΔA746* and *lys2ΔA746*,*NR* alleles in an MMR-deficient (*mlh1Δ*) background. Loss of Mlh1 had, at most, a 1.5-fold effect on QP mutations (Tables S1 and S2), suggesting that the majority of the underlying strand switches are unlikely to occur directly at the replication fork.

### QP mutations are elevated in the absence of nucleotide excision repair

**Figure 4:**
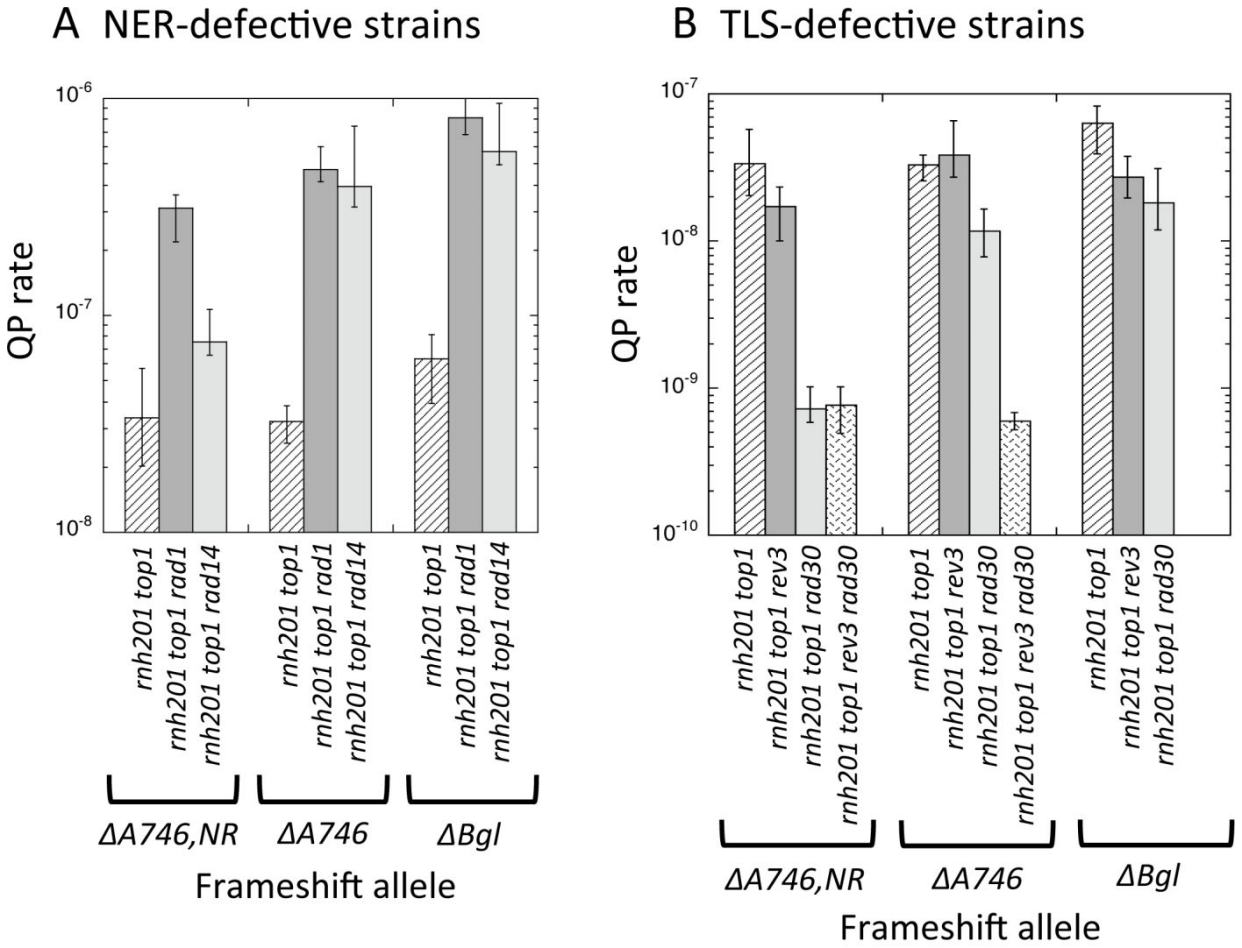
Rates of QP mutations and tandem-repeat deletions at hotspots in NER- or TLS-defective strains. **A.** QP mutation rates in *rnh201Δ top1Δ* strains that are additionally missing the Rad14 or Rad1 component of NER. **B.** QP mutation rates in *rnh201Δ top1Δ* strains that are missing Pol ζ (*rev3*Δ) and/or Pol η (*rad30*Δ).

Transcription-associated mutations that arise in the presence of RNase H2 are elevated when excision repair pathways are disabled and are dependent on the Pol ζ translesion synthesis (TLS) DNA polymerase, suggesting that most arise during the error-prone bypass of DNA damage [34], [36]. To ascertain whether DNA damage is relevant to rNMP-dependent QP mutations, we examined the effect of disabling the nucleotide excision repair (NER) pathway. Rad1, the homolog of mammalian XPF, forms a stable complex with Rad10. Rad1–Rad10 cleaves at the junction between double- and single-strand DNA, and makes one of the incisions that initiates removal of a damage-containing oligonucleotide during NER (reviewed in [37]). This complex additionally has a flap endonuclease activity that removes 3′ single-strand tails that arise during recombination in yeast [38] and has been implicated in repair of hairpin structures formed by CAG trinucleotide repeats [39]. Deletion of *RAD1* in an *rnh201Δ top1Δ* background was associated with an ∼10-fold increase in QP mutations in the each of the frameshift reversion assays (Figure 4A). The rates of Top1-dependent 2–4 bp deletions were not significantly affected by Rad1 loss (Tables S4, S5, S6), however, confirming a fundamentally different mechanism for generating rNMP-dependent QP versus short-deletion events.

To discern whether the effect of Rad1 loss on QP mutations reflects the role of Rad1–Rad10 during NER, we examined the effect of disrupting Rad14, the homolog of mammalian XPA. Rad14 is required for the damage recognition step of NER and its loss eliminates NER in yeast, but not other functions of the Rad1–Rad10 complex. For the QP mutations identified in the *lys2ΔA746* and *lys2ΔBgl* assays, there was no discernable difference between the rates of these events in the *rnh201Δ top1Δ rad14Δ* versus *rnh201Δ top1Δ rad1Δ* strains (Figure 4A). In the *lys2ΔA746,NR* assay, however, the rate of QP mutations in the *rnh201Δ top1Δ rad14Δ* background was only 2.2-fold higher than in the *rnh201Δ top1Δ* background, significantly less than the 9-fold increase observed in the *rnh201Δ top1Δ rad1Δ* background. The QP events identified in the *lys2ΔA746* and *lys2ΔBgl* assays are thus limited by NER, while those detected in the *lys2ΔA746,NR* assay reflect primarily an NER-independent role of Rad1–Rad10.

### Translesion synthesis (TLS) DNA polymerases are important for generating QP mutations

Prior analyses of transcription-associated mutagenesis in the *lys2ΔA746* reversion assay suggest that most events reflect DNA-damage bypass by the Pol ζ TLS DNA polymerase [34], [40]. To examine whether rNMP-dependent QP mutations similarly require Pol ζ activity, we eliminated the Rev3 catalytic subunit of the enzyme in *rnh201Δ top1Δ* strains. In an *rnh201Δ top1Δ rev3Δ* background, the rate of QP mutations in the *lys2ΔA746,NR* or *lys2ΔBgl* assay was reduced ∼2-fold, while that in the *lys2ΔA746* assay was not significantly affected (Figure 4B). Given the small effect of Pol ζ loss, we examined whether Pol η, the other major TLS polymerase in yeast, might be relevant to rNMP-dependent QP mutations. In the *lys2ΔA746,NR* assay, deletion of the Pol η-encoding *RAD30* gene was accompanied by a 50-fold reduction in the QP mutation rate. In the *lys2ΔA746 and lys2ΔBgl* assays, rates of QP mutations rates were reduced only ∼3-fold in a *rad30Δ* background. QP mutations detected using the *lys2ΔA746* allele, however, were completely eliminated upon loss of both Pol ζ and Pol η (*rnh201Δ top1Δ rev3Δ rad30Δ* mutant; reversion of the *lys2ΔBgl* allele was not examined in this background). Altogether, the data indicate that generation of rNMP-dependent QP mutations requires the participation of Pol ζ and/or Pol η, with specific strand-switch events relying on individual TLS polymerases to different extents. Again, this is in contrast to the rates of rNMP- and Top1-dependent deletions in tandem repeats, which are not affected by loss of TLS polymerases.

## Discussion

We previously reported two types of mutations that are specifically elevated by high levels of transcription in yeast. First, removal of uracil that replaces thymine in transcriptionally active DNA creates abasic sites that are bypassed in a mutagenic manner by Pol ζ, resulting in elevated TA to GC transversions [36], [40]. Second, small deletions in tandem repeats reflect Top1 activity, which is important for removing transcription-associated supercoils [19], [20]. Here, we report a third transcription-associated mutation signature comprised of complex sequence changes that extend the complementarity between two arms of a quasi-palindrome (QP). Transcription-associated QP mutations are greatly elevated in an RNase H2-defective (*rnh201Δ*) background and, therefore, are associated with the persistence of some type of RNA∶DNA hybrid. Though both transcription-associated short deletions and QP mutations are elevated in an *rnh201Δ* background, other genetic requirements for these two types of events are distinctly different (summarized in Table 3). Most notably, rNMP-dependent QP mutations are affected by the direction of replication-fork movement and require RNase H1 rather than Top1 activity. In addition, only QP mutations are limited by NER and are dependent on TLS polymerases.

**Table 3:** Summary of genetic requirements for rNMP-dependent mutations.

	rNMP-dependent mutation type	
	QP mutations	2–4 bp deletions
Affected by transcription?	Yes (↑)	Yes (↑)
Affected by Top1 loss?	No	Yes (↓)
Affected by RNase H1 loss?	Yes (↓)	No
Affected by rNMP-restrictive Pol ε?	No	Yes (↓)
Affected by replication direction?	Yes (SAME>OPPO)	No
Affected by MMR loss?	No	No
Affected by NER loss?	Yes (↑)	No
Affected by TLS polymerase loss?	Yes (↓)	No
Affected by HR loss?	No	No

Complex sequence changes at QPs have been most extensively characterized in *E. coli* (reviewed in [30]), but also have been identified in bacteriophage T4 [41] and are found among *TP53* gene mutations in human cancer cells [42]. A template-switch model for QP mutations was first proposed to explain unusual frameshift mutations in the yeast iso-1-cytochrome c gene [43], and we suggest that a similar mechanism is responsible for transcription-associated QP mutations. More recently, QP mutations in yeast have been shown to occur during the repair of DNA double-strand breaks via homologous recombination [44], [45]. Homologous recombination cannot be the source of the transcription-associated QP events observed here, however, as these events are not affected by loss of Rad52 (see Tables S1, S2), a protein essential for recombination in yeast (reviewed in [46]).

In bacterial studies, a strong effect of the direction of replication fork movement on a given QP mutation has been interpreted as evidence that the event primarily reflects either an intra-strand switch during lagging-strand synthesis or a transient inter-strand switch of a nascent leading strand to the lagging-strand template (see Figure 3). In either case, it is assumed that the switch is facilitated by the inherent single-strand character of the lagging-strand template and is precipitated by a block/pause during DNA synthesis. For example, there is a positive correlation between intrinsic DNA polymerase pause sites *in vitro* and QP-associated mutation hotspots in phage T4 [47]. In addition, QP-mediated mutations, but not simple base substitutions or frameshifts, are strongly stimulated by the nucleoside analog 3′-azidothymidine (AZT) in *E. coli*, which causes chain termination and replication stalling when incorporated by DNA polymerases [48]. Because transcription-associated QP mutation rates were much higher in the SAME than OPPO orientation, these events most likely reflect a problem encountered during lagging-strand synthesis. As will elaborated further below, the sensitivity of QP mutations to the direction of replication-fork movement may additionally reflect an orientation-dependent transcription-replication conflict.

In spite of the strong effect of replication direction on transcription-associated QP mutations, these events were not further stimulated by MMR loss and thus are unlikely to occur directly at the fork. Though this is in contrast to the removal of some types of QP mutation intermediates by MMR in *E. coli* [35], we note a similar MMR independence in the specific case of transcription-associated QP mutations [49]. One possible explanation for the MMR independence of transcription-associated QP mutations is that they arise during the filling of gaps that are left behind the fork when processive DNA synthesis is disrupted. Requirements of the yeast TLS polymerases for transcription-associated QP mutations (Figure 4B) would be consistent with such a post-replicative, gap-filling process. Indeed, it has been shown that other types of mutation intermediates introduced by Pol ζ are refractory to correction by the MMR machinery, and hence presumably arise outside the context of a replication fork [25]. Roles of the TLS polymerases during the formation of QP mutations could be in the relatively nonprocessive synthesis required for successive template switches and/or in extending mispaired primer-template termini that arise as a result of a template switch. Pol ζ, for example, is particularly good at extending primer-template mispairs [50].

In contrast to their strong stimulation upon RNase H2 loss, transcription-associated QP mutations are dependent on the presence of RNase H1. RNase H1 requires at least 3–4 contiguous rNMPs to incise the RNA component of RNA∶DNA hybrids, and we consider three possible sources of such hybrids. First, a DNA polymerase could mis-insert consecutive rNMPs during DNA synthesis. Though the major source of stochastic rNMP incorporation into the yeast genome appears to be due to Pol ε [6], expression of a mutant form of Pol ε with a reduced propensity to incorporate rNMPs did not significantly lower the rate of QP mutations. An alternative possibility that we cannot exclude is that one of the yeast TLS polymerases, each of which contributes to QP mutations (Figure 4), might be the source of the consecutive rNMPs that RNase H1 acts on. *E. coli* Pol V, for example, which is in the same polymerase family as yeast Pol η, is characterized by low discrimination between dNTPs and rNTPs [51]. A second source of more extended RNA∶DNA hybrids is the RNA primers used to initiate Okazaki fragments during lagging-strand synthesis. The major pathways for removing RNA primers appear to involve either the successive cleavage of short, Pol δ-displaced 5′ flaps by the Fen1 flap endonuclease or the cleavage of longer strand-displacement flaps by the combined action of Dna2 and Fen1 [5]. This does not exclude, however, an important role for RNases H1 and H2 in Okazaki primer removal in some circumstances, and a specific case relevant to QP mutations might be under high-transcription conditions. Finally, R-loops that form during transcription provide a third source of RNA∶DNA hybrids that are potential substrates of RNase H1. The opposing roles of RNase H1 and H2 in the generation of QP mutations (a reduction and increase upon deletion of *RNH1* and *RNH201*, respectively) suggest that RNase H1 likely degrades most of the RNA component of the relevant RNA∶DNA hybrid, leaving a small number of rNMPs that can only be removed by RNase H2. It is then the persistence of one or a few rNMPs that is assumed to perturb lagging-strand synthesis and eventually precipitate the intra-strand template switch that leads to QP mutations. We note that the type of template switch proposed here would only allow a small number of blocking rNMPs to be bypassed. Longer RNA-DNA hybrids presumably lead to a different type of instability that cannot be detected in our reversion assays.

A final feature of the transcription-associated QP mutations described here is their elevation upon loss of the NER pathway, suggesting that removal of some type of helical distortion or secondary structure limits these events. Helical distortion could reflect DNA damage or the presence of consecutive rNMPs in duplex DNA [52], while the hairpin formed during an intra-strand template switch might be a relevant secondary structure. In addition to a general role for NER in preventing QP mutations, an NER-independent role for Rad1 was evident in the *lys2ΔA746,NR* assay. We speculate that the NER independence reflects the well-known role of Rad1–Rad10 in removing 3′ flaps [38], which may be analogous to the role of 3′ to 5′ single-strand exonucleases in preventing QP mutations in *E. coli* [35]. Whether other endo- or exo-nucleases are relevant to QP mutations in yeast remains to be explored.

**Figure 5:**
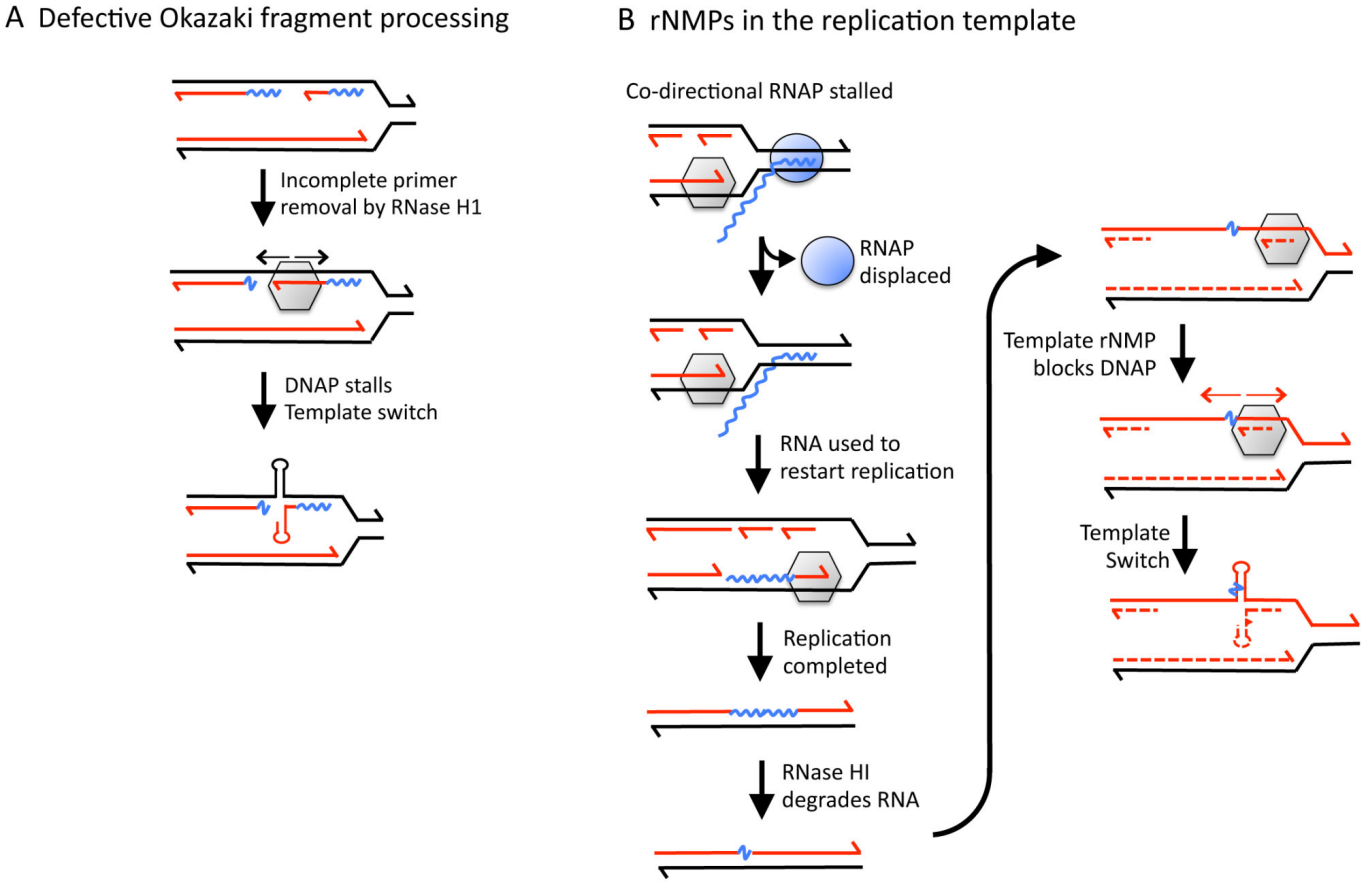
Models for generating rNMP-dependent QP mutations. **A.** A transcription-associated defect in removing Okazaki-fragment primers stalls DNA polymerase (DNAP; gray hexagon), which precipitates an intra-strand template switch. **B.** Following a co-directional collision between DNAP and RNA polymerase (RNAP; blue oval), the replisome uses the transcript as a primer to re-start leading-strand DNA synthesis. Incomplete removal of the transcript-derived RNA places rNMPs in the lagging-strand template at the next round of replication. Stalling of DNAP at the template rNMPs precipitates an intra-strand template switch. Though QP mutations are depicted as being introduced during lagging-strand synthesis that is coupled to the fork, they most likely are generated during post-replicative filling of lagging-strand gaps. Wavy blue lines correspond to RNA.

Based on the genetic requirements of transcription-associated QP mutations, we entertain two different models for how these events might arise in yeast (Figure 5). Central to both models is an involvement of an RNA∶DNA hybrid and an effect of the direction of replication-fork movement. In the first model, replication stalling and subsequent strand misalignment within a QP reflects a deficiency in Okazaki fragment maturation in the absence of RNase H2 (Figure 5A). This would be consistent with the presumptive origin of QP mutations during lagging-strand synthesis and with the apparent site-specificity of these mutations, which could reflect the position of an Okazaki fragment-priming site. There are, for example, additional QPs in the reversion window monitored that should be able to template complex mutations, but corresponding QP mutations were not detected at these positions. The involvement of NER could reflect processing of a hairpin intermediate formed during the template switch, while an NER-independent role of Rad1–10 could reflect removal of unpaired 3′ ends during an attempted template switch [35]. A prediction of this model is that QP mutations should be exacerbated by additional perturbation in Okazaki fragment synthesis and/or processing.

A second model (Figure 5B) is suggested by *in vitro* studies of replication-transcription conflicts using bacterial proteins, in which co-directional collisions disrupt both DNA and RNA synthesis. Significantly, the transcript can be used as a primer to re-start leading-strand replication [53]. Recent studies in *E. coli* have corroborated these findings and suggest that co-directional collisions with backtracked/arrested RNA polymerase are particularly problematic [54]. Following replication re-start, a failure to properly process the primer would lead to transcript-derived rNMPs in the lagging-strand template at the next round of replication. rNMP-provoked replication stalling could trigger formation of an rNMP-containing gap that would need to be filled post-replicatively, leading to the observed QP mutations. For yeast DNA polymerases, the efficiency of rNMP bypass varies depending on the sequence context [55], and this might account for the site-specificity of events. With regard to the role of NER in limiting QP mutations, pausing/stalling of the transcription machinery at damage that is normally removed by NER would be expected to increase co-directional collisions. More frequent collisions should also be promoted by mutations that slow the elongation rate of RNA polymerase or that eliminate anti-backtracking mechanisms. This model could be tested by examining the effect of eliminating transcription elongation factor TFIIS, which also reactivates backtracked RNA polymerase [56].

Ribonucleotides are likely the most prevalent, non-canonical component of genomic DNA and can lead to catastrophic events in the absence of efficient removal [7]. In addition to Top1-generated strand breaks at rNMPs, which lead to short deletions [18] and replication stress [57], the QP mutations described here underscore the importance of efficient rNMP removal by RNase H2 for the maintenance of genome integrity. The more extensive palindromes generated by QP mutations can fold into stable secondary structures such as hairpins and cruciforms, which would further increase the risk of genome instability. Additional studies will be required to understand the molecular mechanism whereby transcription perturbs the processing and/or promotes formation of RNA∶DNA hybrids that lead to QP mutations in yeast.

## Materials and Methods

### Media and growth conditions

Yeast strains were grown at 30°C in non-selective YEP medium (1% yeast extract, 2% peptone; 2% agar for plates) supplemented with 2% dextrose (YEPD) or with 2% each of glycerol and ethanol (YEPGE). *pTET* was maximally activated under these growth conditions, and was repressed by addition of doxycycline (Sigma) to 2 µg/ml. Selective growth was on synthetic, 2% dextrose (SD) medium supplemented with all but one nutrient. Selection of hygromycin- or nourseothricin-resistant transformants was on YEPD plates containing 300 µg/ml hygromycin (Mediatech) or 100 µg/ml nourseothricin (Axxora), respectively.

### Strain constructions

Yeast strains were derived from YPH45 (*MATα ura3-52 ade2-101_oc_ trp1Δ1*). Deletion of the *LYS2* locus and introduction of the *pTET-lys2ΔA746*, *pTET-lys2ΔBgl*, or *pTET-lys2ΔA746,NR* allele near *ARS306* on Chromosome III was previously described [18], [20], [34]. The *pol2-M644L* mutation was introduced by two-step allele replacement using plasmid p173 [17]. Genes were deleted using PCR-generated cassettes containing a selectable marker flanked by ∼60 bp of homology. As appropriate, marker genes flanked by *loxP* sites were deleted using a Cre-expressing plasmid [58].

### Mutation rates and spectra

One-ml YEPGE cultures were inoculated with 250,000 cells and grown for 3 days. Appropriate dilutions were plated on YEPD or SD-Lys plates to determine the total number of cells or the number of Lys^+^ revertants, respectively, in each culture. Data from 10–24 cultures were used to calculate each mutation rate using the method of the median [59], and corresponding 95% confidence intervals were determined as described previously [60]. To construct mutation spectra, we isolated independent Lys^+^ revertants from 0.3-ml YEPGE cultures inoculated from single colonies. Following isolation of genomic DNA, the relevant region of *LYS2* was PCR amplified and sequenced by the Duke University DNA Analysis Facility.

## Supporting Information

Table S1 QP mutations in the *pTET-lys2ΔA746,NR* assay.
(DOCX)

Table S2 QP mutations in the *pTET-lys2ΔA746* assay.
(DOCX)

Table S3 QP mutations in the *pTET-lys2ΔBgl* assay.
(DOCX)

Table S4 2-bp deletions at the TGTCTG hotspot in the *pTET*-*lys2ΔA746,NR* assay.
(DOCX)

Table S5 2-bp deletions at the 6A run hotspot in the *pTET*-*lys2ΔA746* assay.
(DOCX)

Table S6 4-bp deletions at the (AGCT)_2_ hotspot in the *pTET*-*lys2ΔBgl* assay.
(DOCX)

## References

[1] Aguilera A, Garcia-Muse T (2012) R loops: from transcription byproducts to threats to genome stability. Mol Cell 46: 115–124. 22541554 10.1016/j.molcel.2012.04.009

[2] Li X, Manley JL (2006) Cotranscriptional processes and their influence on genome stability. Genes Dev 20: 1838–1847. 16847344 10.1101/gad.1438306

[3] Kim N, Jinks-Robertson S (2012) Transcription as a source of genome instability. Nat Rev Genet 13: 204–214. 22330764 10.1038/nrg3152 PMC3376450

[4] Burgers PM (2009) Polymerase dynamics at the eukaryotic DNA replication fork. J Biol Chem 284: 4041–4045. 18835809 10.1074/jbc.R800062200 PMC2640984

[5] Zheng L, Shen B (2011) Okazaki fragment maturation: nucleases take centre stage. J Mol Cell Biol 3: 23–30. 21278448 10.1093/jmcb/mjq048 PMC3030970

[6] Nick McElhinny SA, Watts BE, Kumar D, Watt DL, Lundstrom EB, et al (2010) Abundant ribonucleotide incorporation into DNA by yeast replicative polymerases. Proc Natl Acad Sci USA 107: 4949–4954. 20194773 10.1073/pnas.0914857107 PMC2841928

[7] Reijns MA, Rabe B, Rigby RE, Mill P, Astell KR, et al (2012) Enzymatic removal of ribonucleotides from DNA is essential for mammalian genome integrity and development. Cell 149: 1008–1022. 22579044 10.1016/j.cell.2012.04.011 PMC3383994

[8] Cerritelli SM, Crouch RJ (2009) Ribonuclease H: the enzymes in eukaryotes. FEBS J 276: 1494–1505. 19228196 10.1111/j.1742-4658.2009.06908.x PMC2746905

[9] Ohtani N, Haruki M, Morikawa M, Crouch RJ, Itaya M, et al (1999) Identification of the genes encoding Mn2+-dependent RNase HII and Mg2+-dependent RNase HIII from *Bacillus subtilis*: classification of RNases H into three families. Biochemistry 38: 605–618. 9888800 10.1021/bi982207z

[10] Chon H, Sparks JL, Rychlik M, Nowotny M, Burgers PM, et al (2013) RNase H2 roles in genome integrity revealed by unlinking its activities. Nucleic Acids Res 41 ((5)): 3130–43. 23355612 10.1093/nar/gkt027 PMC3597693

[11] Cerritelli SM, Frolova EG, Feng C, Grinberg A, Love PE, et al (2003) Failure to produce mitochondrial DNA results in embryonic lethality in RNaseh1 null mice. Mol Cell 11: 807–815. 12667461 10.1016/s1097-2765(03)00088-1

[12] Jeong HS, Backlund PS, Chen HC, Karavanov AA, Crouch RJ (2004) RNase H2 of *Saccharomyces cerevisiae* is a complex of three proteins. Nucleic Acids Res 32: 407–414. 14734815 10.1093/nar/gkh209 PMC373335

[13] Sparks JL, Chon H, Cerritelli SM, Kunkel TA, Johansson E, et al (2012) RNase H2-initiated ribonucleotide excision repair. Mol Cell 47: 980–986. 22864116 10.1016/j.molcel.2012.06.035 PMC3470915

[14] Reagan MS, Pittenger C, Siede W, Friedberg EC (1995) Characterization of a mutant strain of *Saccharomyces cerevisiae* with a deletion of the *RAD27* gene, a structural homolog of the *RAD2* nucleotide excision repair gene. J Bacteriol 177: 364–371. 7814325 10.1128/jb.177.2.364-371.1995 PMC176599

[15] Lazzaro F, Novarina D, Amara F, Watt DL, Stone JE, et al (2012) RNase H and postreplication repair protect cells from ribonucleotides incorporated in DNA. Mol Cell 45: 99–110. 22244334 10.1016/j.molcel.2011.12.019 PMC3262129

[16] Crow YJ, Leitch A, Hayward BE, Garner A, Parmar R, et al (2006) Mutations in genes encoding ribonuclease H2 subunits cause Aicardi-Goutières syndrome and mimic congenital viral brain infection. Nature Genet 38: 910–916. 16845400 10.1038/ng1842

[17] Nick McElhinny SA, Kumar D, Clark AB, Watt DL, Watts BE, et al (2010) Genome instability due to ribonucleotide incorporation into DNA. Nat Chem Biol 6: 774–781. 20729855 10.1038/nchembio.424 PMC2942972

[18] Kim N, Huang SY, Williams JS, Li YC, Clark AB, et al (2011) Mutagenic processing of ribonucleotides in DNA by yeast topoisomerase I. Science 332: 1561–1564. 21700875 10.1126/science.1205016 PMC3380281

[19] Cho JE, Kim N, Li YC, Jinks-Robertson S (2013) Two distinct mechanisms of Topoisomerase 1-dependent mutagenesis in yeast. DNA Repair 12 ((3)): 205–11. 23305949 10.1016/j.dnarep.2012.12.004 PMC3594648

[20] Lippert MJ, Kim N, Cho JE, Larson RP, Schoenly NE, et al (2011) Role for topoisomerase 1 in transcription-associated mutagenesis in yeast. Proc Natl Acad Sci USA 108: 698–703. 21177427 10.1073/pnas.1012363108 PMC3021083

[21] Pommier Y, Pourquier P, Fan Y, Strumberg D (1998) Mechanism of action of eukaryotic DNA topoisomerase I and drugs targeted to the enzyme. Biochim Biophys Acta 1400: 83–105. 9748515 10.1016/s0167-4781(98)00129-8

[22] Sekiguchi J, Shuman S (1997) Site-specific ribonuclease activity of eukaryotic DNA topoisomerase I. Mol Cell 1: 89–97. 9659906 10.1016/s1097-2765(00)80010-6

[23] Greene CN, Jinks-Robertson S (1997) Frameshift intermediates in homopolymer runs are removed efficiently by yeast mismatch repair proteins. Mol Cell Biol 17: 2844–2850. 9111356 10.1128/mcb.17.5.2844 PMC232136

[24] Harfe BD, Jinks-Robertson S (1999) Removal of frameshift intermediates by mismatch repair proteins in *Saccharomyces cerevisiae* . Mol Cell Biol 19: 4766–4773. 10373526 10.1128/mcb.19.7.4766 PMC84275

[25] Lehner K, Jinks-Robertson S (2009) The mismatch repair system promotes Polζ-dependent translesion synthesis in yeast. Proc Natl Acad Sci USA 106: 5749–5754. 19307574 10.1073/pnas.0812715106 PMC2667058

[26] Chen JZ, Qiu J, Shen B, Holmquist GP (2000) Mutational spectrum analysis of RNase H(35) deficient *Saccharomyces cerevisiae* using fluorescence-based directed termination PCR. Nucleic Acids Res 28: 3649–3656. 10982888 10.1093/nar/28.18.3649 PMC110751

[27] Turchi JJ, Huang L, Murante RS, Kim Y, Bambara RA (1994) Enzymatic completion of mammalian lagging-strand DNA replication. Proc Natl Acad Sci USA 91: 9803–9807. 7524089 10.1073/pnas.91.21.9803 PMC44905

[28] Qiu J, Qian Y, Frank P, Wintersberger U, Shen B (1999) *Saccharomyces cerevisiae* RNase H(35) functions in RNA primer removal during lagging-strand DNA synthesis, most efficiently in cooperation with Rad27 nuclease. Mol Cell Biol 19: 8361–8371. 10567561 10.1128/mcb.19.12.8361 PMC84926

[29] Ripley LS (1990) Frameshift mutation: determinants of specificity. Annu Rev Genet 24: 189–213. 2088167 10.1146/annurev.ge.24.120190.001201

[30] Lovett ST (2004) Encoded errors: mutations and rearrangements mediated by misalignment at repetitive DNA sequences. Molecular Microbio 52: 1243–1253. 10.1111/j.1365-2958.2004.04076.x 15165229

[31] Trinh TQ, Sinden RR (1991) Preferential DNA secondary structure mutagenesis in the lagging strand of replication in *E. coli* . Nature 352: 544–547. 1865910 10.1038/352544a0

[32] Rosche WA, Ripley LS, Sinden RR (1998) Primer-template misalignments during leading strand DNA synthesis account for the most frequent spontaneous mutations in a quasipalindromic region in *Escherichia coli* . J Mol Biol 284: 633–646. 9826504 10.1006/jmbi.1998.2193

[33] Yoshiyama K, Higuchi K, Matsumura H, Maki H (2001) Directionality of DNA replication fork movement strongly affects the generation of spontaneous mutations in *Escherichia coli* . J Mol Biol 307: 1195–1206. 11292335 10.1006/jmbi.2001.4557

[34] Kim N, Abdulovic AL, Gealy R, Lippert MJ, Jinks-Robertson S (2007) Transcription-associated mutagenesis in yeast is directly proportional to the level of gene expression and influenced by the direction of DNA replication. DNA Repair 6: 1285–1296. 17398168 10.1016/j.dnarep.2007.02.023 PMC2034516

[35] Viswanathan M, Lacirignola JJ, Hurley RL, Lovett ST (2000) A novel mutational hotspot in a natural quasipalindrome in *Escherichia coli* . J Mol Biol 302: 553–564. 10986118 10.1006/jmbi.2000.4088

[36] Kim N, Jinks-Robertson S (2009) dUTP incorporation into genomic DNA is linked to transcription in yeast. Nature 459: 1150–1153. 19448611 10.1038/nature08033 PMC2730915

[37] Boiteux S, Jinks-Robertson S (2013) DNA repair mechanisms and the bypass of DNA damage in *Saccharomyces cerevisiae* . Genetics 193: 1025–1064. 23547164 10.1534/genetics.112.145219 PMC3606085

[38] Lyndaker AM, Alani E (2009) A tale of tails: insights into the corrdination of 3′ end processing during homologous recombination. Bioessays 31: 315–321. 19260026 10.1002/bies.200800195 PMC2958051

[39] Lin Y, Wilson JH (2012) Nucleotide excision repair, mismatch repair, and R-loops modulate convergent transcription-induced cell death and repeat instability. PloS One 7: e46807. 23056461 10.1371/journal.pone.0046807 PMC3463551

[40] Kim N, Jinks-Robertson S (2010) Abasic sites in the transcribed strand of yeast DNA are removed by transcription-coupled nucleotide excision repair. Mol Cell Biol 30: 3206–3215. 20421413 10.1128/MCB.00308-10 PMC2897580

[41] de Boer JG, Ripley LS (1984) Demonstration of the production of frameshift and base-substitution mutations by quasipalindromic DNA sequences. Proc Natl Acad Sci USA 81: 5528–5531. 6089210 10.1073/pnas.81.17.5528 PMC391739

[42] Greenblatt MS, Grollman AP, Harris CC (1996) Deletions and insertions in the p53 tumor suppressor gene in human cancers: confirmation of the DNA polymerase slippage/misalignment model. Cancer Res 56: 2130–2136. 8616861

[43] Ripley LS (1982) Model for the participation of quasi-palindromic DNA sequences in frameshift mutation. Proc Natl Acad Sci USA 79: 4128–4132. 7051004 10.1073/pnas.79.13.4128 PMC346590

[44] Hicks WM, Kim M, Haber JE (2010) Increased mutagenesis and unique mutation signature associated with mitotic gene conversion. Science 329: 82–85. 20595613 10.1126/science.1191125 PMC4254764

[45] Deem A, Keszthelyi A, Blackgrove T, Vayl A, Coffey B, et al (2011) Break-induced replication is highly inaccurate. PLoS Biol 9: e1000594. 21347245 10.1371/journal.pbio.1000594 PMC3039667

[46] Symington LS (2002) Role of *RAD52* epistasis group genes in homologous recombination and double-strand break repair. Microbiol Mol Biol Rev 66: 630–670. 12456786 10.1128/MMBR.66.4.630-670.2002 PMC134659

[47] Papanicolaou C, Ripley LS (1991) An in vitro approach to identifying specificity determinants of mutagenesis mediated by DNA misalignments. J Mol Biol 221: 805–821. 1942031 10.1016/0022-2836(91)80177-v

[48] Seier T, Zilberberg G, Zeiger DM, Lovett ST (2012) Azidothymidine and other chain terminators are mutagenic for template-switch-generated genetic mutations. Proc Natl Acad Sci USA 109: 6171–6174. 22474374 10.1073/pnas.1116160109 PMC3341039

[49] Yoshiyama K, Maki H (2003) Spontaneous hotspot mutations resistant to mismatch correction in *Escherichia coli*: transcription-dependent mutagenesis involving template-switching mechanisms. J Mol Biol 327: 7–18. 12614604 10.1016/s0022-2836(03)00089-5

[50] Prakash S, Prakash L (2002) Translesion DNA synthesis in eukaryotes: a one- or two-polymerase affair. Genes Dev 16: 1872–1883. 12154119 10.1101/gad.1009802

[51] McDonald JP, Vaisman A, Kuban W, Goodman MF, Woodgate R (2012) Mechanisms employed by *Escherichia coli* to prevent ribonucleotide incorporation into genomic DNA by Pol V. PLoS Genet 8: e1003030. 23144626 10.1371/journal.pgen.1003030 PMC3493448

[52] Wahl MC, Sundaralingam M (2000) B-form to A-form conversion by a 3′-terminal ribose: crystal structure of the chimera d(CCACTAGTG)r(G). Nucleic Acids Res 28: 4356–4363. 11058136 10.1093/nar/28.21.4356 PMC113134

[53] Pomerantz RT, O'Donnell M (2008) The replisome uses mRNA as a primer after colliding with RNA polymerase. Nature 456: 762–766. 19020502 10.1038/nature07527 PMC2605185

[54] Dutta D, Shatalin K, Epshtein V, Gottesman ME, Nudler E (2011) Linking RNA polymerase backtracking to genome instability in *E. coli* . Cell 146: 533–543. 21854980 10.1016/j.cell.2011.07.034 PMC3160732

[55] Watt DL, Johansson E, Burgers PM, Kunkel TA (2011) Replication of ribonucleotide-containing DNA templates by yeast replicative polymerases. DNA Repair 10: 897–902. 21703943 10.1016/j.dnarep.2011.05.009 PMC3147116

[56] Wind M, Reines D (2000) Transcription elongation factor SII. BioEssays 22: 327–336. 10723030 10.1002/(SICI)1521-1878(200004)22:4<327::AID-BIES3>3.0.CO;2-4 PMC3367499

[57] Williams JS, Smith DJ, Marjavaara L, Lujan SA, Chabes A, et al (2013) Topoisomerase 1-mediated removal of ribonucleotides from nascent leading-strand DNA. Mol Cell 49 ((5)): 1010–5. 23375499 10.1016/j.molcel.2012.12.021 PMC3595360

[58] Gueldener U, Heinisch J, Koehler GJ, Voss D, Hegemann JH (2002) A second set of *loxP* marker cassettes for Cre-mediated multiple gene knockouts in budding yeast. Nucleic Acids Res 30: e23. 11884642 10.1093/nar/30.6.e23 PMC101367

[59] Lea DE, Coulson CA (1949) The distribution of the numbers of mutants in bacterial populations. J Genet 49: 264–285. 24536673 10.1007/BF02986080

[60] Spell RM, Jinks-Robertson S (2004) Determination of mitotic recombination rates by fluctuation analysis in *Saccharomyces cerevisiae*. In: Waldman AS, editor. Genetic Recombination: Reviews and Protocols. Totowa, NJ: Humana Press. pp. 3–12. 10.1385/1-59259-761-0:003 14769952

