# Supplementary material for: RNA∶DNA Hybrids Initiate Quasi-Palindrome-Associated Mutations in Highly Transcribed Yeast DNA
Source: PLoS Genet. 2013 Nov 7;9(11):e1003924. doi: 10.1371/journal.pgen.1003924 (PMC3820800; doi:10.1371/journal.pgen.1003924)
Supplement: Table S1 — QP mutations in the pTET-lys2ΔA746,NR assay. (DOCX) [file pgen.1003924.s001.docx]

**Table S1. QP mutations in the *pTET-lys2ΔA746,NR* assay**

| Relevant genotype | Orientation | Lys^+^ rate (X 10^-10^)  (95% CI) | No. of each QP type observed | | Fraction of mutations at QP | QP rate X 10^-10^ [relative to *rnh201* SAME] |
| --- | --- | --- | --- | --- | --- | --- |
|  |  |  | 1 | 2 |  |  |
| WT, low txn | SAME | 9.5  (8.6 – 10.4) |  |  | 0/90 | <0.11 |
| *rnh201*, low txn | SAME | 15.3  (11.7 – 17.9) | 2 |  | 2/91 | 0.34 |
| WT | SAME | 161  (118 – 216) |  |  | 0/86 | <1.9 |
| WT | OPPO | 150  (106 – 209) |  |  | 0/91 | <1.6 |
| *rnh201* | SAME | 1470  (1210 – 1710) | 6 | 5 | 11/48 | 337 [1.0] |
| *rnh201* | OPPO | 566  (397 – 691) |  |  | 0/46 | <12 |
| *rnh201 top1* | SAME | 812  (486 – 1370) | 10 | 10 | 20/48 | 338 [1.0] |
| *rnh201 top1* | OPPO | 410  (258 – 937) | 4 |  | 4/46 | 36 |
| *rnh201 rnh1* | SAME | 1030  (613 – 2140) |  |  | 0/88 | <12 [0.4] |
| *rnh201 top1*  *pol2 M644L* | SAME | 553  (496 – 732) | 30 | 21 | 51/92 | 307 [0.91] |
| *rnh201 top1 mlh1* | SAME | 775  (488 – 866) | 22 | 13 | 35/79 | 343 [1.0] |
| *rnh201 rad1* | SAME | 6970  (5150 – 7620) | 9 | 10 | 19/47 | 2820 [8.4] |
| *rnh201 top1 rad1* | SAME | 7500  (5290 – 8640) | 18 | 21 | 39/94 | 3110 [9.2] |
| *rnh201 top1 rad14* | SAME | 2330  (2040 – 3320) | 18 | 12 | 30/93 | 752 [2.2] |
| *rnh201 top1 rev3* | SAME | 369  (218 – 502) | 23 | 19 | 42/91 | 170 [0.50] |
| *rnh201 top1 rad30* | SAME | 210  (170 – 302) | 3 |  | 3/88 | 7.2 [0.02] |
| *rnh201 top1 rad30 rev3* | SAME | 120  (77.5 – 143) | 4 |  | 4/63 | 7.6 [0.02] |
| *rnh201 rad52* | SAME | 2350  (2180 – 3240) | 6 | 8 | 14/81 | 406 [1.2] |
| *rnh201 rnh1 rad1* | SAME | 2210  (1510 – 4140) | 2 | 3 | 5/90 | 123 [0.37] |

Lys^+^ revertants were isolated under high-transcription conditions unless noted otherwise. The classes of QP mutation types are defined in Figure 1C. CI, confidence interval.
