# Supplementary material for: RNA∶DNA Hybrids Initiate Quasi-Palindrome-Associated Mutations in Highly Transcribed Yeast DNA
Source: PLoS Genet. 2013 Nov 7;9(11):e1003924. doi: 10.1371/journal.pgen.1003924 (PMC3820800; doi:10.1371/journal.pgen.1003924)
Supplement: Table S2 — QP mutations in the pTET-lys2ΔA746 assay. (DOCX) [file pgen.1003924.s002.docx]

**Table S2. QP mutations in the *pTET-lys2ΔA746* assay**

| Relevant genotype | Orientation | Lys^+^ rate (X 10^-10^)  (95% CI) | No. of each QP/cpx type observed | | | | Fraction of mutations at QP | QP rate X 10^-10^ [relative to *rnh201* SAME] |
| --- | --- | --- | --- | --- | --- | --- | --- | --- |
|  |  |  | 1 | 2 | 3 | 4 |  |  |
| WT, low txn | SAME | 18.4  (15.1 – 27.6) |  |  |  |  | 0/73 | <0.25 |
| *rnh201*, low txn | SAME | 33.4  (21.2 – 59.3) |  |  |  |  | 0/91 | <0.37 |
| WT | SAME | 428  (335 – 674) |  |  |  |  | 0/117 | <3.7 |
| WT | OPPO | 415  (353 – 572) |  |  |  |  | 0/115 | <3.6 |
| *rnh201* | SAME | 3530  (2700 – 4090) | 9 | 2 |  |  | 11/78 | 499 [1.0] |
| *rnh201* | OPPO | 1490  (1410 – 2070) | 2 |  |  | 1 | 3/93 | 48 |
| *rnh201 top1* | SAME | 1420  (1120 – 1670) | 18 |  |  | 2 | 20/87 | 326 [0.65] |
| *rnh201 top1* | OPPO | 711  (590 – 830) | 3 |  |  | 1 | 4/91 | 31 |
| *rnh201 rnh1* | SAME | 2530  (1650 – 5710) | 1 |  |  |  | 1/88 | 29 [0.06] |
| *rnh201 top1*  *pol2 M644L* | SAME | 982  (758 – 1070) | 26 | 1 |  | 3 | 30/94 | 313 [0.63] |
| *rnh201 top1 mlh1* | SAME | 2830  (2510 – 3690) | 14 | 1 |  | 1 | 16/94 | 482 [0.96] |
| *rnh201 rad1* | SAME | 16300  (13600 – 20300) | 25 | 1 | 2 | 7 | 35/91 | 6269 [13] |
| *rnh201 top1 rad1* | SAME | 10900  (9960 – 13800) | 27 | 2 | 3 | 7 | 39/90 | 4723 [9.5] |
| *rnh201 top1 rad14* | SAME | 7320  (5890 – 13900) | 34 | 4 | 7 | 4 | 49/91 | 3942 [7.7] |
| *rnh201 top1 rev3* | SAME | 1110  (782 – 1880) | 26 | 1 |  | 3 | 30/86 | 387 [0.78] |
| *rnh201 top1 rad30* | SAME | 798  (537 – 1130) | 9 |  |  | 4 | 13/89 | 117 [0.23] |
| *rnh201 top1 rad30 rev3* | SAME | 469  (403 – 808) | 1 |  |  |  | 1/78 | 6.0 [0.01] |
| *rnh201 rad52* | SAME | 3640  (2550 – 5970) | 2 | 1 |  |  | 3/63 | 173 [0.35] |
| *rnh201 rad1 rnh1* | SAME | 2860  (2550 – 4370) | 1 |  |  | 1 | 2/94 | 61 [0.12] |

Lys^+^ revertants were isolated under high-transcription conditions unless noted otherwise. The classes of QP mutation types are defined in Figure 1B. CI, confidence interval.
