# Supplementary material for: RNA∶DNA Hybrids Initiate Quasi-Palindrome-Associated Mutations in Highly Transcribed Yeast DNA
Source: PLoS Genet. 2013 Nov 7;9(11):e1003924. doi: 10.1371/journal.pgen.1003924 (PMC3820800; doi:10.1371/journal.pgen.1003924)
Supplement: Table S3 — QP mutations in the pTET-lys2ΔBgl assay. (DOCX) [file pgen.1003924.s003.docx]

**Table S3. QP mutations in the *pTET-lys2ΔBgl* assay**

| Relevant genotype | Orientation | Lys^+^ rate (X 10^-10^)  (95% CI) | No. of each QP type observed | | | | Fraction of mutations at QP | QP rate X 10^-10^ [relative to *rnh201* SAME] |
| --- | --- | --- | --- | --- | --- | --- | --- | --- |
|  |  |  | 1 | 2 | 3 | 4 |  |  |
| WT, low txn | SAME | 31.9  (26.3 – 47.3) |  |  |  |  | 0/73 | <0.44 |
| *rnh201*, low txn | SAME | 68.8  (44.3 – 120) |  |  |  |  | 0/84 | <0.82 |
| WT | SAME | 784  (586 – 1310) |  |  |  |  | 0/77 | <10 |
| WT | OPPO | 719  (569 – 798) |  |  |  |  | 0/74 | <9.7 |
| *rnh201* | SAME | 3060  (2300 – 5790) | 17 |  | 3 | 6 | 26/92 | 865 [1.0] |
| *rnh201* | OPPO | 2740  (2320 – 4390) |  |  |  |  | 0/93 | <29 |
| *rnh201 top1* | SAME | 2080  (1390 – 2990) | 21 |  |  | 6 | 27/89 | 631 [0.73] |
| *rnh201 top1* | OPPO | 1570  (1170 – 1800) |  |  |  |  | 0/81 | <19 |
| *rnh201 rnh1* | SAME | 3180  (2280 – 4820) | 2 |  |  |  | 2/85 | 75 [0.09] |
| *rnh201 rad1* | SAME | 9550  (8350 – 13100) | 13 | 5 | 19 | 15 | 52/94 | 5280 [6.1] |
| *rnh201 top1 rad1* | SAME | 14100  (12200 – 20000) | 5 | 16 | 14 | 19 | 54/93 | 8190 [9.5] |
| *rnh201 top1 rad14* | SAME | 8840  (7720 – 14800) | 12 | 12 | 12 | 24 | 60/93 | 5700 [6.6] |
| *rnh201 top1 rev3* | SAME | 653  (475 – 892) | 29 | 2 |  | 4 | 35/85 | 269 [0.31] |
| *rnh201 top1 rad30* | SAME | 1090  (720 – 1890) | 14 |  |  | 1 | 15/91 | 180 [0.21] |

Lys^+^ revertants were isolated under high-transcription conditions unless noted otherwise. The classes of QP mutation types are defined in Figure 1A. CI, confidence interval.
