# Supplementary material for: RNA∶DNA Hybrids Initiate Quasi-Palindrome-Associated Mutations in Highly Transcribed Yeast DNA
Source: PLoS Genet. 2013 Nov 7;9(11):e1003924. doi: 10.1371/journal.pgen.1003924 (PMC3820800; doi:10.1371/journal.pgen.1003924)
Supplement: Table S4 — 2-bp deletions at the TGTCTG hotspot in the pTET-lys2ΔA746,NR assay. (DOCX) [file pgen.1003924.s004.docx]

**Table S4. 2-bp deletions at the TGTCTG hotspot in the *pTET*-*lys2ΔA746,NR* assay**

| Relevant  genotype | Orientation | Lys^+^ rate X 10^-10^  (95% CI) | Fraction of 2-bp deletions | 2-bp deletion rate X 10^-10^  [relative to *rnh201* SAME] |
| --- | --- | --- | --- | --- |
| WT, low txn | SAME | 9.5  (8.6 – 10.4) | 1/90 | 0.11 |
| *rnh201*, low txn | SAME | 15.3  (11.7 – 17.9) | 41/91 | 6.9 |
| WT | SAME | 161  (118 – 216) | 0/86 | <1.9** |
| WT | OPPO | 150  (106 – 209) | 0/91 | <1.6** |
| *rnh201* | SAME | 1470  (1210 – 1710) | 26/48 | 796 [1.0] |
| *rnh201* | OPPO | 566  (397 – 691) | 32/46 | 394 |
| *rnh201 top1* | SAME | 812  (486 – 1370) | 0/48 | <18 [0.02] |
| *rnh201 top1* | OPPO | 410  (258 – 937) | 0/46 | <8.9 |
| *rnh201 rnh1* | SAME | 1030  (613 – 2140) | 48/88 | 562 [0.71] |
| *rnh201 rad1* | SAME | 6970  (5150 – 7620) | 5/47 | 741 [0.93] |
| *rnh201 rad52* | SAME | 2350  (2180 – 3240) | 27/81 | 783 [0.98] |
| *rnh201 rnh1 rad1* | SAME | 2210  (1510 – 4140) | 46/90 | 1129 [1.4] |

Lys^+^ revertants were isolated under high-transcription conditions unless noted otherwise. CI, confidence interval.
