# Supplementary material for: RNA∶DNA Hybrids Initiate Quasi-Palindrome-Associated Mutations in Highly Transcribed Yeast DNA
Source: PLoS Genet. 2013 Nov 7;9(11):e1003924. doi: 10.1371/journal.pgen.1003924 (PMC3820800; doi:10.1371/journal.pgen.1003924)
Supplement: Table S5 — 2-bp deletions at the 6A run hotspot in the pTET-lys2ΔA746 assay. (DOCX) [file pgen.1003924.s005.docx]

**Table S5. 2-bp deletions at the 6A run hotspot in the *pTET*-*lys2ΔA746* assay**

| Relevant  genotype | Orientation | Lys^+^ rate X 10^-10^  (95% CI) | Fraction of 2-bp deletions | 2-bp deletion rate X 10^-10^  [relative to *rnh201* SAME] |
| --- | --- | --- | --- | --- |
| WT, low txn | SAME | 18.4  (15.1 – 27.6) | 0/73 | <0.25 |
| *rnh201*, low txn | SAME | 33.4  (21.2 – 59.3) | 26/91 | 9.5 |
| WT | SAME | 428  (335 – 674) | 17/117 | 62.2 |
| WT | OPPO | 415  (353 – 572) | 15/115 | 54.1 |
| *rnh201* | SAME | 3530  (2700 – 4090) | 27/78 | 1220 [1.0] |
| *rnh201* | OPPO | 1490  (1410 – 2070) | 43/93 | 689 |
| *rnh201 top1* | SAME | 1420  (1120 – 1670) | 0/87 | <16.3 [<0.01] |
| *rnh201 top1* | OPPO | 711  (590 – 830) | 1/91 | 7.8 |
| *rnh201 rnh1* | SAME | 2530  (1650 – 5710) | 38/88 | 1090 [0.89] |
| *rnh201 rad1* | SAME | 16300  (13600 – 20300) | 5/91 | 896 [0.73] |
| *rnh201 rad52* | SAME | 3640  (2550 – 5970) | 17/63 | 982 [0.80] |

Lys^+^ revertants were isolated under high-transcription conditions unless noted otherwise. CI, confidence interval.
