# Supplementary material for: RNA∶DNA Hybrids Initiate Quasi-Palindrome-Associated Mutations in Highly Transcribed Yeast DNA
Source: PLoS Genet. 2013 Nov 7;9(11):e1003924. doi: 10.1371/journal.pgen.1003924 (PMC3820800; doi:10.1371/journal.pgen.1003924)
Supplement: Table S6 — 4-bp deletions at the (AGCT)2 hotspot in the pTET-lys2ΔBgl assay. (DOCX) [file pgen.1003924.s006.docx]

**Table S6. 4-bp deletions at the (AGCT)_2_ hotspot in the *pTET*-*lys2ΔBgl* assay**

| Relevant  genotype | Orientation | Lys^+^ rate X 10^-10^  (95% CI) | Fraction of 4-bp deletions | 4-bp deletion rate X 10^-10^  [relative to *rnh201* SAME] |
| --- | --- | --- | --- | --- |
| WT, low txn | SAME | 31.9  (26.3 – 47.3) | 5/73 | 2.2 |
| *rnh201*, low txn | SAME | 68.8  (44.3 – 120) | 69/84 | 56.5 |
| WT | SAME | 784  (586 – 1310) | 1/77 | 10.2 |
| WT | OPPO | 719  (569 – 798) | 1/74 | 9.7 |
| *rnh201* | SAME | 3060  (2300 – 5790) | 35/92 | 1160 [1.0] |
| *rnh201* | OPPO | 2740  (2320 – 4390) | 51/93 | 1500 |
| *rnh201 top1* | SAME | 2080  (1390 – 2990) | 0/89 | <23 [0.02] |
| *rnh201 top1* | OPPO | 1570  (1170 – 1800) | 0/81 | <19 |
| *rnh201 rnh1* | SAME | 3180  (2280 – 4820) | 29/85 | 1085 [0.94] |
| *rnh201 rad1* | SAME | 9550  (8350 – 13100) | 9/94 | 914 [0.80] |

Lys+ revertants were isolated under high-transcription conditions unless noted otherwise. CI, confidence interval.
